# The Role of Comprehensive Hybrid Imaging in Identification of Plaque Rupture and Ostial Stent Placement: Case Report

**DOI:** 10.1016/j.jscai.2025.103814

**Published:** 2025-07-29

**Authors:** Natali Sorajja, Khady N. Fall, Akiko Maehara, Megha Prasad

**Affiliations:** a Department of Medicine, Columbia University Irving Medical Center/NewYork-Presbyterian Hospital, New York, New York; b Department of Medicine, Albert Einstein College of Medicine, Bronx, New York; c Cardiovascular Research Foundation, New York, New York

**Keywords:** case report, imaging, ostial lesion, percutaneous coronary intervention, plaque rupture

## Abstract

A 52-year-old male smoker with multiple risk factors (hypertension, hyperlipidemia, type 2 diabetes mellitus, and end-stage renal disease treated with dialysis), exertional angina, and inferior ischemia was referred for angiography. He was found to have an ostial right coronary artery (RCA) lesion that was thought to be calcified based on angiography. Hybrid imaging was performed with intravenous ultrasound (IVUS) and optical coherence tomography (OCT) using the Novasight Hybrid IVUS-OCT System catheter (Conavi Medical), which helped identify plaque rupture as opposed to calcification. OCT, with its superior resolution, demonstrated plaque rupture that was difficult to appreciate on IVUS. IVUS was used to mark the ostium of the RCA and precisely place a stent in the ostial RCA.

## Clinical case

A 52-year-old male smoker with hypertension, hyperlipidemia, type 2 diabetes mellitus, and end-stage renal disease treated with dialysis, exertional angina, and inferior ischemia was referred for angiography.

## Procedure

**Figure 1:**
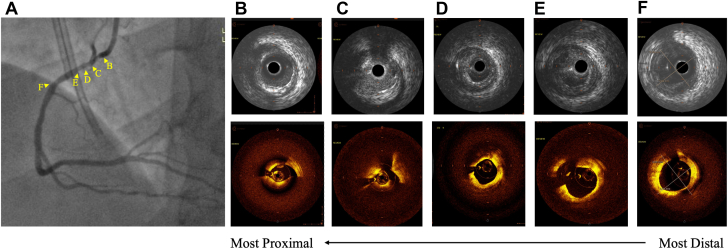
**Before percutaneous coronary intervention.** (A) Angiogram of right coronary artery (RCA) lesion; intravenous ultrasound (top) and optical coherence tomography (bottom) visualizations of (B) ostial RCA, (C) conus branch, (D) intraplaque hemorrhage and wire artifact, (E) plaque rupture cavity and wire artifact, and (F) distal reference.

**Figure 2:**
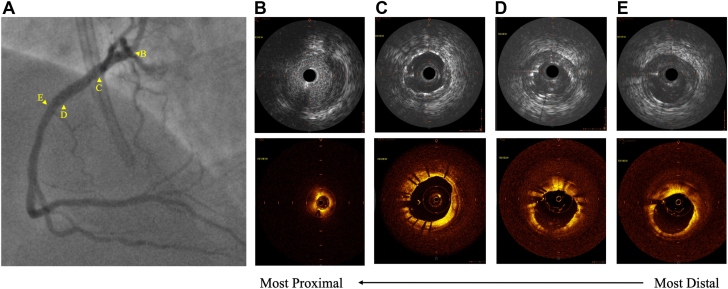
**After percutaneous coronary intervention.** (A) Angiogram of right coronary artery (RCA) lesion; intravenous ultrasound (top) and optical coherence tomography (bottom) visualizations of (B) proximal edge of stent struts of ostial RCA (demonstrating no extension into the aorta), (C) minimal stent area of 9.40 mm^2^, (D) distal stent edge struts and wire artifact, and (E) the distal reference.

Angiography showed a critical, likely calcified stenosis of ostial/proximal right coronary artery (RCA). Supplemental Videos 1 and 2 demonstrate preangiography imaging and a prehybrid imaging, respectively. The image acquisition (pullback) speed for the prehybrid imaging run was 25 mm/s. However, hybrid imaging with intravenous ultrasound (IVUS) and optical coherence tomography (OCT) was subsequently performed, which revealed plaque rupture and no significant calcification. Distal reference area was 15.11 mm^2^, with a mean distal reference diameter of 3.93 mm. The length from the distal reference to the ostial RCA was 31.3 mm. Plaque rupture was more readily apparent on OCT than that on IVUS, with area of stenosis equaling 82%. Intraplaque hemorrhage was also present, with prepercutaneous coronary intervention (PCI) minimum lumen area of 2.79 mm^2^ and plaque burden of 70%. The conus branch was visually identified, as well as the ostial RCA past the conus branch. Figure 1 shows all pre-PCI imaging, including IVUS, OCT, and angiogram. The RCA lesion was dilated with a 3.0-mm × 12.0-mm balloon, and a 3.5-mm × 33.0-mm stent was placed at the ostium. IVUS and “jog-mode” on the hybrid imaging system was used to accurately mark the ostium. Supplemental Video 3 shows the lesion postangiography. Post-PCI distal vessel diameter was 3.88 mm, with the distal stent edge struts also visualized. Post-PCI minimal stent area was measured to be 9.40 mm^2^, with distal reference minimum lumen area of 6.7 mm^2^, thus confirming stent expansion of over 100%. Visualization of the 2 proximal stent edge struts at the ostial RCA also demonstrated no protrusion into the aorta. Figure 2 shows all post-PCI imaging, including IVUS, OCT, and angiogram.

## Discussion

Identification of plaque rupture may be challenging with angiography and IVUS alone; hybrid imaging allowing concomitant OCT may be useful both clinically and for educational purposes.^1^ Previous research has demonstrated that in acute coronary syndrome, IVUS alone can fail to recognize acute lesions such as plaque rupture thrombus; therefore, hybrid imaging may increase ease of diagnosis.^2^ Plaque rupture may be especially challenging to identify on IVUS for inexperienced imagers, in part due to the lower spatial resolution of IVUS and morphology of plaque rupture.^2^^,^^3^ In this case, the use of hybrid imaging allows identification of plaque rupture with a frame-by-frame correlation, which serves as an important teaching tool. This case illustrates a subtler appearance of plaque rupture on IVUS than on OCT. When compared with IVUS, OCT allows superior visualization of residual thrombus within the plaque rupture cavity. From a technical standpoint, hybrid imaging was particularly helpful in allowing the operator to leverage IVUS for optimal ostial stent placement, which might have been challenging with an OCT only system.

Furthermore, the original false-positive finding of calcified stenosis on angiography, with no significant calcification on IVUS/OCT, is very rare. The sensitivity of IVUS and OCT in detecting calcium has been found to be much higher in comparison with angiographic calcium detection, with recent research showing angiography detecting 40.2% of calcified lesions, IVUS detecting 82.7%, and OCT detecting 76.8%.^4^, ^5^, ^6^ We speculate that our case of this rare instance is due to lipidic plaque masking underlying calcium and that the deep location and thick nature of the calcium in this lesion further contributed toward this finding. Furthermore, the ECLIPSE trial also demonstrated that angiographic calcium estimation can be operator dependent.^6^ This is another example of how hybrid imaging utilization can overcome limitations of other imaging measures, such as angiography, and can provide more precise assessments.

Thus, this procedure was optimized by having both the superior resolution of OCT and the ability to mark the ostium with IVUS. Access to both IVUS and OCT in a single catheter, therefore, has important clinical implications, especially in patients with high-risk plaques and/or ostial disease.^7^ Moreover, hybrid imaging can be a useful educational tool to improve an operator’s imaging skills.
